# Frags2Drugs: A Novel In Silico Fragment-Based Approach to the Discovery of Kinase Inhibitors

**DOI:** 10.3390/ph19020308

**Published:** 2026-02-12

**Authors:** Gautier Peyrat, Colin Bournez, Pascal Krezel, José-Manuel Gally, Stéphane Bourg, Samia Aci-Sèche, Pascal Bonnet

**Affiliations:** Université d’Orléans, CNRS, ICOA, UMR 7311, Orléans, Francestephane.bourg@cnrs.fr (S.B.)

**Keywords:** kinase research, fragment growing, fragment-based ligand design (FBLD), network graph, protein kinase inhibitor

## Abstract

**Background/Objectives**: Fragment-based approaches in the field of drug discovery and design have been widely developed and employed in both academia and industry. We present here an innovative in silico fragment-based drug design approach aimed at designing new inhibitors in the ATP-binding site of protein kinases. **Methods**: This tool, named Frags2Drugs (F2D), relies on a three-dimensional fragment library obtained from co-crystallized ligands. This library is stored in a graph-oriented database containing the required information to link fragments together. F2D builds every possible molecule that fits into the given cavity on a minute scale. Molecules are then filtered to keep those presenting the best predicted affinity. Several specific molecular filters can be applied, including protein kinase inhibitor-like filters. **Results**: We validated our method by reconstructing existing co-crystallized ligands and known kinase inhibitors. In this study, we provide several examples of its use to retrieve known or design new type I, type I1/2, type II, and macrocyclic inhibitors on several protein kinases. **Conclusions**: We have developed an in silico fragment-based ligand design tool able to identify novel kinase inhibitors by growing any scaffolds positioned in the ATP-binding site..

## 1. Introduction

In recent decades, fragment-based drug design (FBDD) has emerged to become a major approach in the discovery of new chemical compounds [1]. The first success from FBDD dates from 2011, with U.S. Food and Drug Administration (FDA) approval of vemurafenib (Zelboraf), a drug targeting the mutated protein kinase BRAF V600E [2]. Nowadays, several drugs approved or in advanced clinical trials have been discovered by using FBDD approaches [3,4]. Recent examples are erdafetinib (Balversa) [5], developed by Janssen and Astex and approved by the FDA in 2019; capivasertib (Truqap), an AKT kinase inhibitor approved in 2023 and developed by AstraZeneca; and sevabertinib, the last approved drug targeting the kinase domain of ERBB2.

Fragments are an interesting alternative to traditional compounds used in high-throughput screenings: they are smaller (usually < 300 Da) and cover a larger chemical space [6]. Once identified during a screening campaign, fragment hits are optimized by medicinal chemists using, when available, structural approaches such as X-ray crystallography or Nuclear Magnetic Resonance (NMR) spectroscopy. Computational chemistry may also play a substantial role in this process. Multiple methods have been recently developed in the field of in silico FBDD [7] that can be classified into three main categories: growing, linking, and merging.

Growing methods are the most commonly used, by adding new fragments sequentially from an initial fragment, usually called the seed. Software programs such as AutoGrow [8,9,10], FOG [11], and Open-Growth [12] are based on this strategy. Other tools use atoms rather than fragments to optimize the seed, such as LEGEND [13] and Genstar [14]. However, atom-based methods remain less employed nowadays. The growth is usually performed in the binding site, using the three-dimensional coordinates of the original hit found experimentally or by docking methods. Available space surrounding the attachment atom and physicochemical properties of the residues in the binding site are important features for the optimization of the seed.

The second strategy, fragment linking, starts from multiple fragments rather than from a unique seed, with the aim of connecting them together directly or using a linker. This method is equivalent to the experimental Structure Activity Relationships (SAR) by NMR approach [15]. The protein target needs to present distinct pockets in its binding site, allowing the starting fragments to bind at different key interaction sites. The fragment linking method is notably employed in the tools LUDI [16], LEA3D [17], GANDI [18], and Fragment-Shuffling [19]. More recently, DeLinker [20], a deep learning tool, was developed to generate linkers between fragments using three-dimensional information.

The third and perhaps less employed strategy is fragment merging. It relies on the superimposition of multiple fragments presenting overlapping structural groups. Common structural features of the fragments are combined to create novel inhibitors.

One of the main challenges of computational FBDD is to design synthesizable compounds. Therefore, some software applications, such as LigBuilder [21] or PINGUI [22], include an estimation of the synthetic accessibility in their algorithms with the help of chemical rules applied during the growth and/or with retrosynthetic analysis.

**Figure 1:**
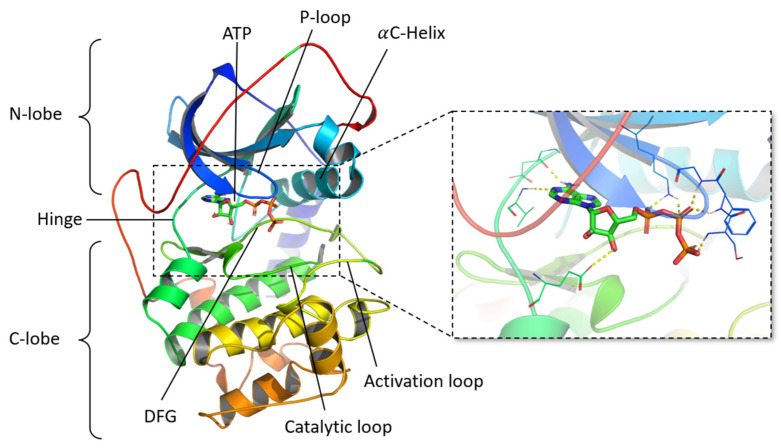
Cartoon representation of a protein kinase crystallographic structure (PDB ID 1ATP, chain E) with a focus on the ATP binding site, highlighting the interactions between ATP and the residues of the catalytic site (yellow dotted lines). N-lobe: N-terminal lobe; C-lobe: C-terminal lobe; ATP: Adenosine triphosphate; P-loop: phosphate-binding loop; DFG: Asp-Phe-Gly.

Most of the FBDD programs spend a major part of their calculation time in the systematic exploration of the available space in the binding site, as well as in energy minimization of the generated compounds to find an equilibrium position. At the same time, the number of experimental protein structures with co-crystallized ligand is continuously expanding, providing more available structural information and data to be harnessed. From this observation, it appears relevant to develop a method exploiting these data so that the time dedicated to cavity exploration and energy minimization is considerably reduced. Frags2Drugs (F2D), presented in this study, uses fragments from co-crystallized ligands or from docking experiments and searches for possible combinations between them to construct new molecules. F2D is a method based on both fragment growing and linking strategies. The growing is performed by linking fragments already in the binding site and placing them close to each other. F2D requires 3D fragments, generated from the fragmentation of crystal structures of ligands, in close vicinity, to create covalent bonds while keeping their initial position. During this work, we were particularly interested in a specific class of protein, named protein kinases, having common ATP binding sites. Protein kinases are enzymes that catalyze the transfer of a γ-phosphate group of ATP to a protein substrate or the same protein during autophosphorylation. They are critical for intracellular transduction, and deregulation of a member of this protein family may lead to diverse diseases, including cancer, autoimmune, or neurological troubles [23]. Their determining role in transduction pathways continues to make kinases targets of major interest for pharmaceutical industries now and over the last decades [24,25]. From a structural point of view, protein kinases share a common catalytic domain composed of two distinct lobes: an N-terminal lobe (N-lobe) and a C-terminal lobe (C-lobe) connected by a hinge region. The cleft between these two lobes is the binding site of the cofactor, ATP, which forms hydrogen bonds with the hinge region, stabilizing its position in the catalytic site (Figure 1). Most kinase inhibitors in development or already on the market target the ATP binding site [26], but other druggable pockets are known, such as the allosteric site or the back pocket [27]. For computational chemists working on protein kinases, the wealth of information coming from structural data is a strong opportunity: 7786 structures of human kinase domains are deposited in the Research Collaboratory for Structural Bioinformatics (RCSB) Protein DataBank [28] (PDB, https://www.rcsb.org/, accessed on 15 December 2025).

Protein kinase inhibitors (PKIs) are divided into seven types depending on their binding modes [29]. The first three types are non-covalent ATP competitive inhibitors. Type I inhibitors bind in DFG-in kinase conformation, type I1/2 in DFG-in conformation with access to an adjacent hydrophobic pocket, and type II inhibitors bind kinases in DFG-out conformation, lying from the ATP site to the adjacent allosteric pocket. Other types are allosteric inhibitors (types III and IV), bivalent inhibitors (type V), and covalent inhibitors (type VI).

## 2. Results

### 2.1. F2D Implementation

F2D is an FBDD tool relying on 3D experimental data. Indeed, fragments obtained from 3D crystallographic structures of protein kinase-ligand complexes fill the database used by F2D to generate novel ligands.

**Figure 2:**
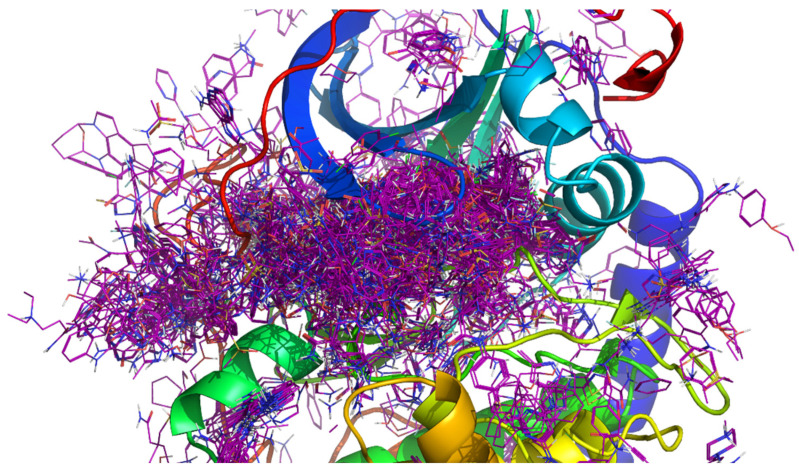
Graphical representation of all fragments after superimposition of all protein kinases on a common reference (PDB ID 1ATP, chain E). Fragments are colored in purple.

To create the fragment library used in F2D, we first extracted protein kinase crystallographic structures available from the RCSB protein databank by only keeping the kinase domain. We then performed a customized structural alignment of all crystal structures on the reference structure: the first crystallized structure of the protein kinase bound with ATP [30] (PDB ID 1ATP, chain E, Figure 2). Once aligned, structures were cleaned to remove ions, co-factors, crystallization agents [31], and solvent molecules. Ligands were afterward extracted and fragmented using fragmentation tools (see Materials and Methods) while keeping their 3D coordinates, so their placements in the aligned active site were kept and recorded in a database.

A recent study applied a similar approach to the one used for the creation of our F2D database. The Kinase–Ligand Interaction Fingerprints and Structure database (KLIFS) is composed of an alignment of 85 key residues from kinase binding sites [32]. Based on fragments from KLIFS, co-crystallized ligands were collected and fragmented. This collection of fragments, called KinFragLib [33], has been used to generate novel kinase inhibitors. Because it is based solely on KLIFS, this work is limited to kinase–ligand complexes only from human and mouse species. In addition, fragments obtained in KinFragLib are restricted to only type I and I1/2 inhibitors. In F2D, we extended the database to all species available in the RSCB PDB, which allowed the creation of type II inhibitors in addition to types I and I1/2.

The aim of F2D is to link several fragments together in a target protein kinase to create potential new bioactive molecules. The structure of the protein kinase used by F2D could be a holo form where the co-crystallized ligand is removed, an apo form for which no co-crystallized ligand was identified, or a homology model of an unknown crystal structure of a protein kinase. We decided to store each fragment in a graph database along with its 3D position and the information of the neighboring fragments. Indeed, a graph network is particularly suited to store information on connectable entities and, thus, perfectly fulfills the aim of F2D with linkable fragments. By browsing a graph of connected fragments, those fragments can be linked together to form a possible PKI.

### 2.2. Creation of the 3D Fragment Network

**Figure 3:**
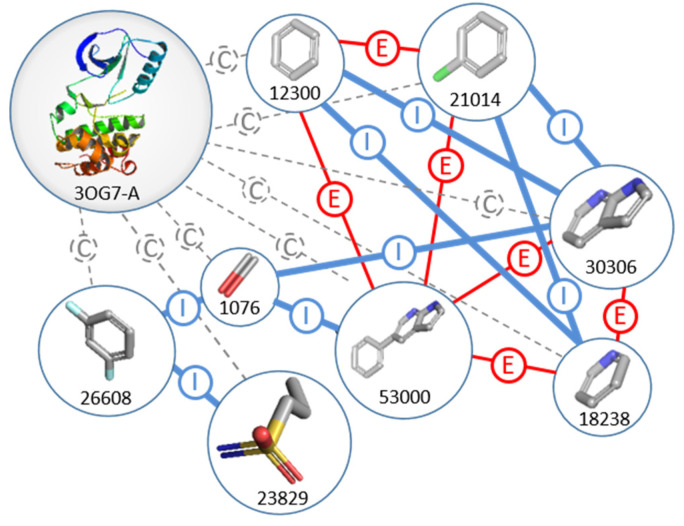
Representation of the F2D graph database, focused on fragments obtained after fragmentation of vemurafenib. Nodes are 3D fragment structures and 3D target proteins. Edges are colored according to their type: exclusion (E in red), inclusion (I in blue), and compatibility (C in gray). Fragment IDs are mentioned.

Fragments are represented by nodes in a 3D fragment network and are linked by edges. The graph database of F2D is composed of two types of nodes (fragment and protein) linked by three types of edges: inclusion, exclusion, and compatibility. A sample of the F2D graph database with only nodes and edges from the PDB ID 3OG7 and vemurafenib fragments is shown in Figure 3.

The first step to create the graph database consists of calculating the relations between all the fragments. These relations belong to two types for ligand information only: inclusion or exclusion, and one type for protein and ligand information: compatibility. An inclusion relation means that the two considered fragments satisfy all the following conditions to bind together: an atom pair exists, one from each fragment, having incomplete valences and separated by a distance that meets a given allowed interval. Moreover, the out-of-plane (OOP), dihedral, and torsion angles with the direct neighbors also meet predefined allowed intervals. The definition of allowed intervals is based on MMFF94 [34,35,36,37,38] force field values by taking into account the type and the hybridization of both atoms involved in the link.

**Figure 4:**
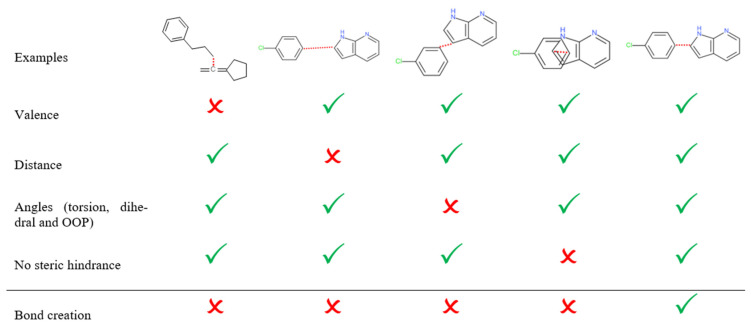
Bond formation conditions between two fragments. Molecules are drawn in 2D for visual purposes, but they are stored in 3D in the F2D database. In each example, the red dash lines represent the potential bond between both fragments. OOP: out of plane. Red crosses correspond to invalid bonds, green ticks correspond to valid bond.

If the two atoms of each fragment are too close or if there is a non-realistic angle value between them, an exclusion relation is established between them. It means they cannot be linked. Furthermore, they cannot belong to the same built molecule, even if they have inclusion relations with a common intermediate fragment. If the two atoms are too far from each other, no relation is established between them. These rules are summarized in Figure 4. Moreover, to avoid suggesting unfeasible molecules to medicinal chemists, additional connection rules were added, such as prohibiting the creation of undesired substructures, detailed in Materials and Methods.

The second step of the creation of the graph database is the calculation of compatibility relations between the fragments and all the residues in the binding site of the protein kinase target. Fragments are compatible if their atoms fulfill an allowed distance between each atom of the fragment and an atom of the residue of the binding site. When this condition is met, there is no steric hindrance between the fragment and the protein; the fragment is kept; otherwise, it is removed for the fragment linking step.

In the graph database, additional information is encoded in nodes and edges. The attributed molecular weight is assigned to fragment nodes and used to stop the addition of new fragments during molecule generation. The inclusion edges, between fragment nodes that can be linked, contain complementary information. When an inclusion relation can be established between two fragments, each linkable atom’s information is saved, as well as the type of bond (simple, double, or triple) that will be made. Linking fragments from molecules bound to different protein kinases could result in a little distortion of their angles and distance compared to reference values from the force field MMFF94 [34]. We thus defined four parameters to set the thresholds of allowed distortions: three parameters for angles (dihedral, out of plane, and torsion) and one for distance. The lower the parameter values, the less the generated molecules are distorted.

### 2.3. Molecular Generation and Filtering Process

F2D molecular generation is initialized with a fragment, referred to as the seed, and a PDB structure or a homology model of the targeted kinase. In a drug design project, a seed can be drawn in 2D by a medicinal chemist, then added, in 3D, to an F2D database after a molecular docking. This is useful when a medicinal chemist is working on a novel scaffold. Starting from the seed, F2D uses only fragments compatible with the chosen protein kinase. Then, F2D searches all possible paths through bonding relations and extracts the corresponding graph. To save time in further treatments, this graph is split into two subgraphs: the Graph of inclusions (Gi) of nodes linked by inclusion relations and the Graph of exclusions (Ge) with only exclusion relations between nodes. In Gi, F2D browses all possible paths starting from the seed. For each obtained path, the tool checks if Ge contains one of its edges (meaning an exclusion relation and therefore an incompatibility for fragments belonging to the same molecule). If so, the corresponding path is discarded. Once all possible paths are obtained and validated, the construction of the corresponding molecules is performed.

In order to build molecules of reasonable size, F2D stops the growing step until a molecular weight (MW) of 650 Da is reached. The value is based on the upper limit of MW published PKI (Table 1).

At this stage, several thousand molecules can be created, which is too large for all to be synthesized. However, due to the presence of the same fragment belonging to the same ligands but co-crystallized in different crystal structures (like imatinib bound to Abl or Src, PDB ID 2HYY, 2OIQ, 3G6G, and 4CSV), we remove duplicate molecules based on their 3D conformation. We removed molecules having a Root Mean Square Deviation (RMSD) ≤ 1.0 Å between two identical created molecules. This also means that several conformations of the same molecule are kept if their RMSD is greater than 1.0 Å. Next, we implemented additional filters to keep only compounds that fulfill the PKI chemical space. Some filters already exist, especially for oral drug assessment, such as Lipinski’s Rule of Five (Ro5) [39]. According to this rule, a compound will probably not be efficiently absorbed orally if there are at least two violations of the following constraints: MW ≤ 500, calculated logP (ClogP) ≤ 5, number of hydrogen bond acceptors (HBA) ≤ 10, number of hydrogen bond donors (HBD) ≤ 5. Other physicochemical properties, such as topological polar surface area (TPSA) and number of rotatable bonds (NRB), can also be used to predict a reliable oral bioavailability (TPSA ≤ 140 Å2 and NRB ≤ 10) [40]. By analyzing a large collection of PKIs in the protein kinase inhibitor database (PKIDB) [41,42], we identified the upper and lower limits of various molecular physicochemical parameters (Table 1). We also allowed the violation of 2 of the 8 parameters, so molecules created by F2D are kept if they fulfil at least 6 of these parameters.

Compounds with undesired substructures are then filtered out. Once all the PKI-like filters are applied, the results obtained by F2D are downloadable as an SDF file.

In F2D, the molecules are built directly inside the binding site of the protein kinase target. No minimization or conformational optimization is performed during the building process of 3D molecules inside the cavity. Therefore, to make sure that the molecules adopt a reliable conformation, we docked each created molecule in the binding site. After the docking step, the resulting docking poses are ranked in ascending order of their difference in RMSD with the original created molecule. F2D only keeps conformations of the created molecule if the RMSD is less than 2.5 Å, and the conformation with the lowest RMSD is first provided to the user.

**Figure 5:**
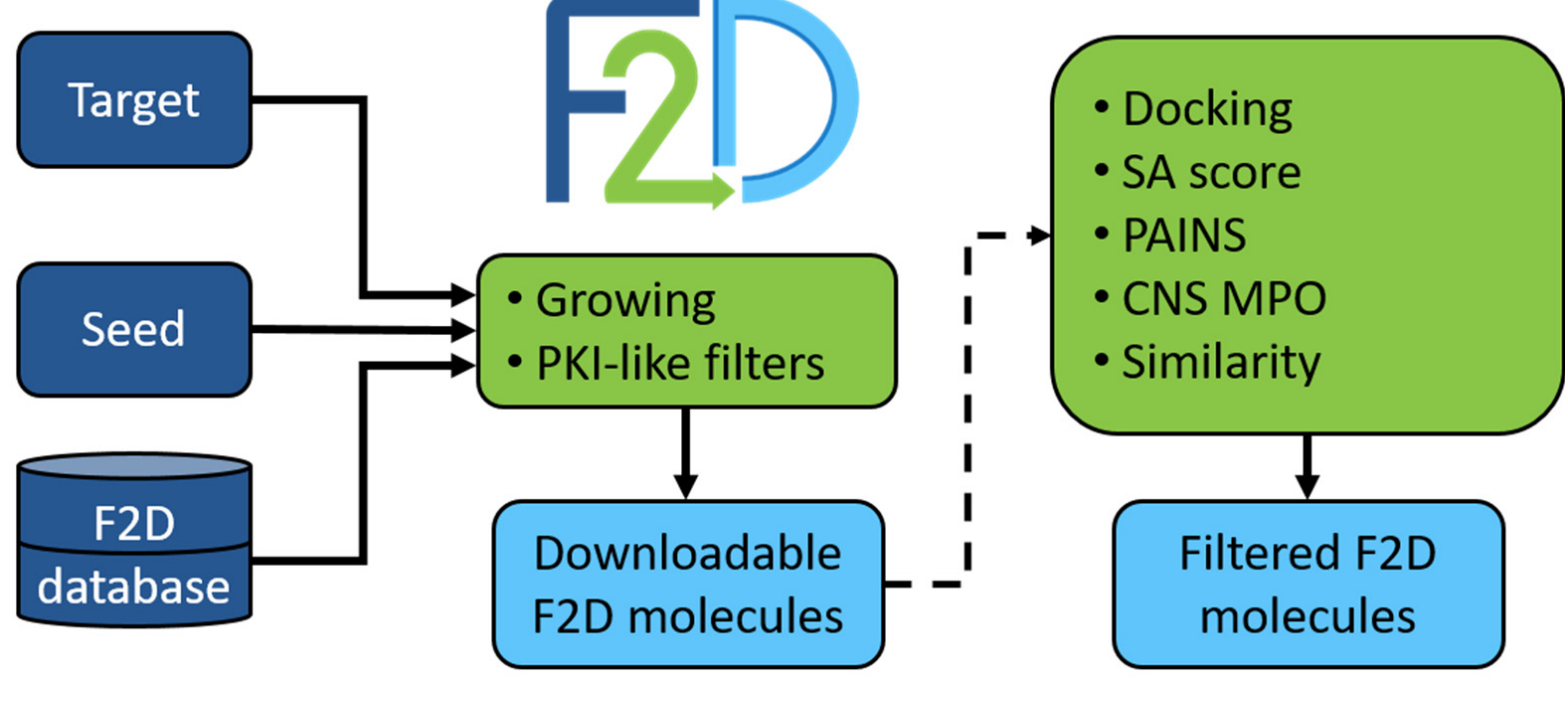
General F2D workflow. Three inputs are required to run F2D: a target and a seed (which can be either picked from the F2D database or a new docked fragment), and the F2D database. Inputs are colored in dark blue, F2D functions are colored in light green, and outputs are colored in light blue. The dotted arrow indicates optional filtering steps.

Other post-processing tools were developed to reduce and optimize the number of suggested compounds by F2D, but as they depend on the target to which F2D is applied, they are non-compulsory. For instance, molecules having Pan-Assay Interference Compounds (PAINS) [43] substructures can be identified and removed. To evaluate the synthetic feasibility of created molecules, a synthetic accessibility (SA) score [44] is calculated. Molecules obtaining an SA score < 4 are thus favored. In the same way, the central nervous system multiparameter optimization (CNS MPO) score [45] estimates the ability of molecules to pass through the blood–brain barrier (BBB). An upper threshold value of 4 is set to keep CNS-like compounds. Finally, as the objectives of F2D are to find active and innovative compounds, we implemented a similarity search to compounds with known bioactivities in the ChEMBL [46] database, and to purchasable compounds available in ZINC [47] or Ambinter [48]. We extended this similarity search to protein kinase inhibitors in clinical trials available in PKIDB. Figure 5 summarizes the general F2D workflow. At the end of the process, an SDF file containing all the information about the origin of each fragment forming each molecule is returned.

After the implementation of the F2D molecule generation process, we validated the F2D methodology.

### 2.4. Validation of F2D Method

**Figure 6:**
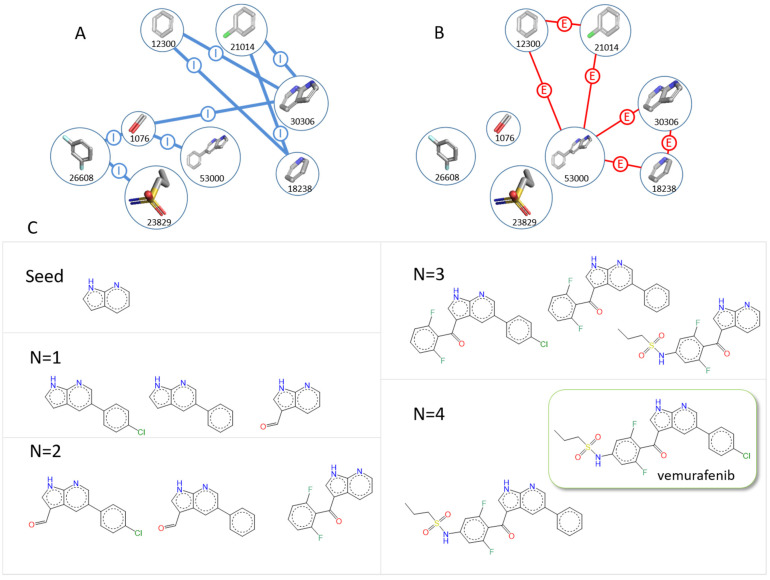
Example of validation of the reconstruction of vemurafenib (PDB ID 3OG7). (**A**) Detailed of a graph of inclusion (Gi) relations between nodes (blue). (**B**) Detailed of a graph of exclusion (Ge) relations between nodes (red). (**C**) Representation of created molecules for each addition (*N*) of a new fragment, starting from the seed 7-azaindole. For better visualization, molecules are shown in 2D.

As a first validation step of the F2D methodology, we aimed to build 3218 co-crystallized structures of ligands bound to a protein kinase from the RCSB protein databank. Each ligand was fragmented to create a 3D fragment network. Then, F2D started the growing process from the fragment bound to the hinge region. The tool succeeded in reconstructing 88.13% of the 3218 co-crystallized kinase inhibitors. As an example of F2D successes, Figure 6 shows the reconstruction of vemurafenib starting from the 7-azaindole seed.

A total of 382 ligands could not be reconstructed by the program. However, after in-depth analysis, these failures are not due to the F2D process itself but to the ligand fragmentation. Indeed, we observed in several cases the absence of fragments in the database, required to build the final ligand. Fragmentation methods employed in F2D sometimes lead to fragments with molecular weights > 300 Da, and those fragments are removed from the F2D database.

After this first validation step, we applied F2D to several protein kinase targets to ensure its ability to find innovative and active compounds. Firstly, we searched for BCR-ABL inhibitors, secondly for BRAF V600E inhibitors, thirdly for type I and type II inhibitors of MELK, and, finally, for ALK macrocyclic inhibitors. These four examples of F2D are detailed below.

#### 2.4.1. Discovery of BCR-ABL Inhibitors

**Figure 7:**
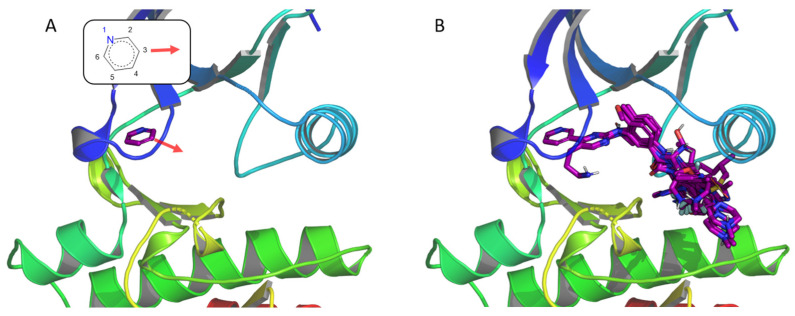
Example of F2D applied to BCR-ABL (PDB ID 2HYY, chain (**A**)). (**A**) Initial position of the seed from the co-crystallized ligand, the red arrow indicates the starting atom and the growing direction. 2D representation of the fragment is shown. (**B**) Representation of the 50 molecules obtained in the active site of BCR-ABL.

Breakpoint Cluster Region-Abelson oncogene (BCR-ABL) is a member of the tyrosine kinase family, and its deregulation may lead to chronic myeloid leukemia (CML) [49]. Before discovering its involvement in CML, classical anticancer treatments were given to patients, such as busulfan and hydroxyurea [50]. Due to the cytotoxic effects of these compounds, recombinant interferon-alfa (rIFN-α) has also been employed [51]. However, thanks to increasing knowledge of CML and with the availability of structural data [52], the first inhibitor targeting specifically BCR-ABL, imatinib [53], reached the market in 2001, becoming the reference treatment of CML. Due to the resistance to this drug observed in several patients, a second generation of Abl inhibitors has been developed since then, such as dasatinib, nilotinib, and bosutinib [54]. As BCR-ABL is today one of the most studied kinases, we chose this protein kinase for the first F2D study. The structure used to assess the F2D process is the wild-type ABL kinase domain co-crystallized with imatinib (PDB ID 2HYY, chain A). The pyridine group of imatinib, which is bound to the hinge region, was selected as a seed (Figure 7A). From this seed, we aimed to grow toward the hydrophobic pocket of the cavity. Therefore, after a visual inspection of the seed positioned in the 3D crystal structure, we selected the C atom in the meta position relative to the *N* atom as a unique starting point, which corresponds to position 3 of the seed (Figure 7).

F2D generated 2595 molecules and clustered them (RMSD ≤ 1.0 Å) to keep 1128 of them, 514 of which fulfil PKI-like filters as described in Table 1. Then, we applied the filtering process of the F2D workflow. Among the 514 molecules, 505 do not have a PAINS substructure, and of those 505 molecules, 495 molecules have a SA score < 4. The 495 remaining molecules were docked to check if the combination of several fragments from different crystallized ligands could form favorable interactions with the active site of Abl (PDB ID 2HYY). A total of 138 molecules have an RMSD value lower than 2.5 Å. Unsurprisingly, as it is present in many Abl inhibitors, we found the N-phenyl-4-(3-pyridyl)pyrimidin-2-amine moiety as a common substructure.

**Figure 8:**
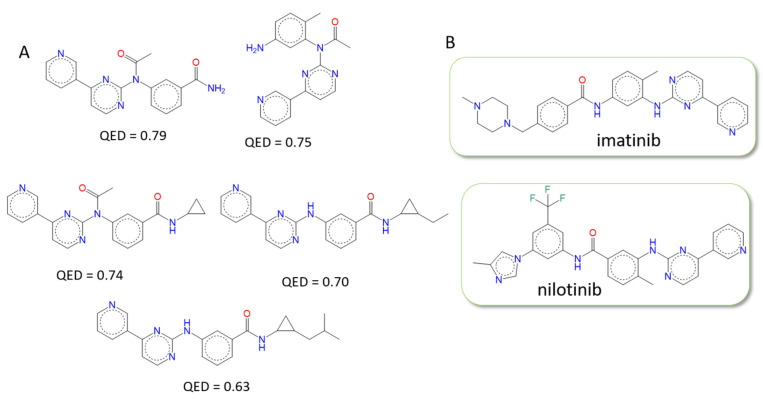
Structures of molecules obtained by generating molecules with F2D on BCR-ABL from the pyridine moiety as a seed. (**A**) Top 5 of the 50 new molecules sorted by QED score. (**B**) Two PKI found among the results: imatinib and nilotinib.

We then searched for similar molecules in the ChEMBL, ZINC, Ambinter, and PKIDBs to check the novelty of the built compounds and found 50 new molecules among the 138 retained. The 2D structures of these compounds are provided in S1, and the 3D superimposition inside the binding site is shown in Figure 8B. The 5 best molecules, according to the Quantitative Estimate of Drug-likeness (QED) score [55], are shown in Figure 8A. Their QED scores range from 0.63 to 0.79. They have a higher probability of becoming a hit than the other molecules generated by F2D. The expertise of medicinal chemists would be required to further optimize these molecules.

The 88 other molecules present similarities with at least one published molecule, including imatinib, nilotinib, radotinib, flumatinib, bafetinib, and masitinib, by considering a Tanimoto coefficient (Tc) of 0.7. Two of them correspond to the exact structure of known drugs found by F2D: imatinib and nilotinib (Figure 8B).

#### 2.4.2. Discovery of BRAF V600E Inhibitors

B–Rapidly Accelerated Fibrosarcoma (BRAF) is a serine/threonine kinase and a member of the RAF family [56]. BRAF is mutated in several cancers [57], the common mutation V600E is mostly related to melanoma [58]. Vemurafenib (Zelboraf), targeting BRAF V600E [2], was found by using structural data from sorafenib (Nexavar), a CRAF inhibitor [59] having low inhibition on BRAF V600E. As vemurafenib is the first approved drug derived from FBDD, we chose BRAF as a second target example for assessing F2D. The structure provided to F2D is chain A of PDB ID 3OG7, which is the structure of BRAF V600E co-crystallized with vemurafenib. The 7-azaindole bound to the hinge, already used in the validation example of F2D (Figure 6), was selected as a seed (Figure S1A), but here the complete F2D database was used in the process.

We obtained 69,868 unique PKI-like molecules. We applied all F2D filters, removed molecules with PAINS substructures (305 molecules), and molecules having a SA score < 4, leading to 12,378 final molecules. After docking, only 525 docked molecules showed an RMSD value lower than 2.5 Å compared to the molecules generated by F2D.

Among the 525 molecules, 510 found no match by similarity search (Tc > 0.7) in ChEMBL, ZINC, Ambinter, or PKIDBs (Figure S1B). The only common substructure they share is the pyrrolo[2,3-b]pyridine seed. The higher number of new molecules found on the BRAF V600E target compared to BCR-ABL can be explained by the four growing atoms in the 7-azaindole seed. A total of 15 molecules among the 510 have a QED ≥ 0.75 and are shown in Figure S2.

In total, 510 molecules were new, and 15 molecules have at least one similar molecule found in one of the four databases, including vemurafenib, which was also reconstructed by F2D. The 2D structures of the 510 new molecules are depicted in the Supplementary Information.

#### 2.4.3. Discovery of MELK Inhibitors

Maternal Embryonic Leucine zipper Kinase (MELK) is another serine/threonine kinase, a member of the CAMK family [60]. Its deregulation seems to be implicated in many cancers [61], and this subject was challenged in the literature [62]. Type I and type II inhibitors have already been discovered for MELK [63], also from FBDD approaches [64,65].

##### Type I MELK Inhibitors

We first searched for new type I inhibitors of MELK. The target and the seed were chosen from the PDB ID 4UMQ structure, which is MELK co-crystallized with 3-{5-[(3-hydroxy-5-methoxyphenyl) amino]-2-(phenylcarbamoyl) phenoxy} propan-1-aminium. This structure has a DFG motif and an αC-helix in the in-conformation. The seed positioned in the protein active site and the starting growing directions are shown in Figure S3A.

87,961 molecules were generated, reduced to 86,669 molecules without PAINS substructures, and only 3934 molecules with a SA score < 4. After the docking step, this number decreased to 41 molecules presenting the closest docking pose with a RMSD value < 2.5 Å. Their 3D structures are presented in Figure S3B. Visualization of those molecules confirms they are type I inhibitors occupying only the ATP-binding pocket and not the allosteric pocket.

All the 41 molecules, selected after applying F2D filters, are new molecules. They have no similar compounds in ChEMBL, Ambinter, ZINC, or PKIDB. Molecules with the best QED score are drawn in Figure S4. The 41 molecules are provided in the Supplementary Information.

##### Type II MELK Inhibitors

Secondly, we focused on the discovery of new type 2 inhibitors of MELK. We selected the PDB ID 4UMT, which is the structure of MELK co-crystallized with 1-(4-{[3-(isoquinolin-7-yl)prop-2-yn-1-yl]oxy}-2-methoxybenzyl)piperazinediium [65]. This conformation of the target has a DFG-in motif and an out-form of the αC helix. We started the fragment growing from the 3-(isoquinolin-7-yl)prop-2-yn-1-ol moiety seed (Figure S5A).

We found 63 unique PKI-like molecules. Then, we applied F2D filters, and 3 molecules were removed because they contained PAINS substructures, and 18 molecules were kept with SA scores < 4. After docking, 14 molecules had an RMSD value < 2.5 Å. From these 14 molecules, 6 molecules were similar (Tc > 0.7) to at least one compound present in ChEMBL, and none to the other databases. Five of the 6 ChEMBL molecules were synthesized and biologically tested on MELK [65]. Their respective ChEMBL IDs and MELK activity are CHEMBL3355060 (MELK IC50 = 24 μM), CHEMBL3355061 (MELK IC50 = 5.1 μM), CHEMBL3355062 (MELK IC50 = 0.65 μM), CHEMBL3355063 (MELK IC50 = 0.52 μM), CHEMBL3355066 (MELK %inhibition = 52% at 300 μM), and CHEMBL215098 (not tested on MELK).

The 8 other molecules have low similarity (Tc < 0.7) to the compounds in the ChEMBL, ZINC, Ambinter, or PKIDBs. Figure S5B represents one of these 8 new molecules. Their QED scores vary from 0.45 to 0.73. The 2D structures of the molecules with the best QED score are shown in Figure S6. The 2D structures of these 8 new molecules are given in the Supplementary Information.

The type II inhibitors found on MELK provide better selectivity on crystallized human protein kinases than type I inhibitors.

#### 2.4.4. Discovery of Macrocyclic Inhibitors Targeting ALK

Macrocycles represent an interesting class of compounds able to inhibit challenging targets [66,67]. Even if there are more macrocyclic natural products on the market than synthetic macrocycles, the latter provide a better kinase affinity and selectivity than other compounds [66]. Fragmentation and generation of macrocycles is not straightforward in in silico FBDD. Fragmentation methods often fail to accurately cut the correct bonds in macrocycles. To manage this, we used several fragmentation methods on the crystallized PKI used to generate the F2D fragment database. However, some parts of the ligands still could not be fragmented and provided fragments with a molecular weight > 300 Da, which were deleted as found also during the validation step of F2D (see above validation of F2D method). Regardless, F2D was able to generate macrocycles from fragments contained in the graph database.

We focused on Anaplastic Lymphoma Kinase (ALK) target and its macrocyclic inhibitor lorlatinib. ALK is a tyrosine kinase belonging to the insulin receptor subfamily implicated in the nervous system’s development and function [68]. Chromosomal translocations involving ALK result in several fusion proteins identified in cancers [69,70,71] as well as the amplification and mutations of the full ALK gene [72,73,74,75].

**Figure 9:**
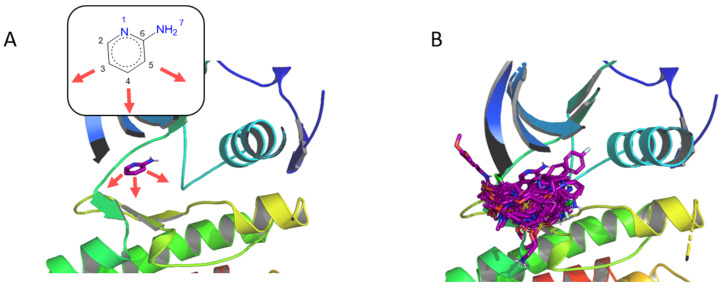
Example of F2D on ALK (PDB ID 4CLI, chain A). (**A**) Initial position of the aminopyridine seed in the active site, the red arrows indicate the starting atoms and the growing directions. 2D representation of the fragment is shown. (**B**) New macrocyclic molecules obtained in the active site of ALK.

Several ALK inhibitors have already been described, such as crizotinib [76] and lorlatinib, which is a macrocycle targeting wild-type or L1196M mutant ALK [77]. We selected the PDB ID 4CLI as the target for F2D. We chose the 3 carbon atoms in ortho, meta, and para positions from the amine of the aminopyridine moiety seed to start the growing (Figure 9A).

Macrocycles are characterized by a ring composed of at least 12 atoms [66]. However, to maximize the number of macrocycles after F2D execution, we selected molecules with at least 11 atoms in their largest rings. We obtained 592 molecules having macrocycles with lengths from 11 to 15 atoms. A total of 35 molecules were removed because of PAINS substructures. Because the synthesis of macrocycles is more complex than for other molecules, we increased the threshold of the SA score used to retain the more feasible ones. Thus, we obtained 174 molecules having an SA score < 5. After the docking experiment, 153 macrocycles show an RMSD value < 2.5 Å between the docked poses and the molecules generated by F2D.

No similar compound with a Tc > 0.7 was found in the ChEMBL, Ambinter, ZINC, or PKIDBs, leading to 153 new macrocycles identified by F2D (Figure 9B).

**Figure 10:**
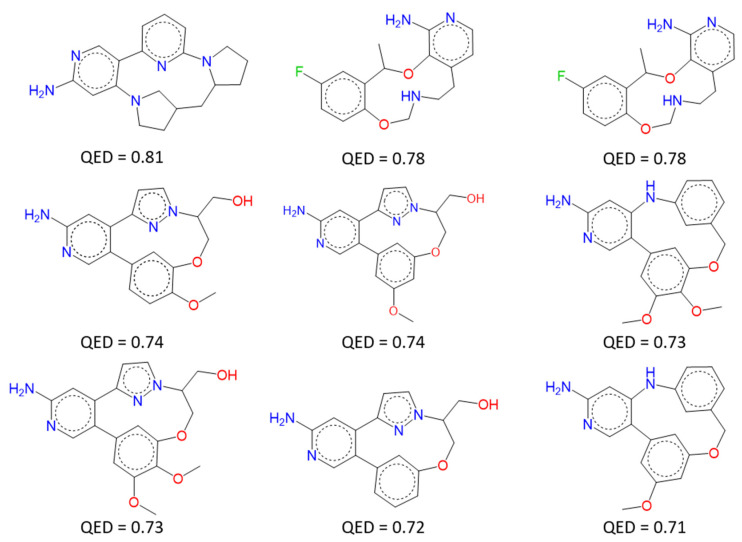
Structures of the 9 macromolecules having the highest QED scores generated with F2D on ALK (PDB ID 4CLI) from an amino-pyridine moiety seed.

**Figure 11:**
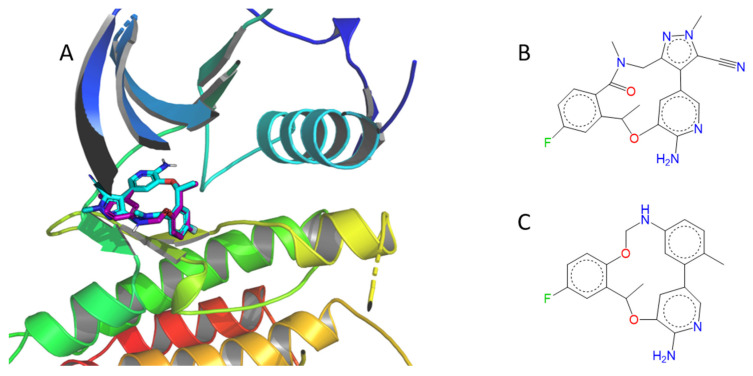
Comparison between lorlatinib and the most similar expected inhibitor found in Frags2Drugs. (**A**) 3D structures of co-crystallized lorlatinib (in cyan) and the F2D inhibitor (in purple) bound in the ALK active site (PDB ID 4CLI). (**B**) 2D structure of lorlatinib. (**C**) 2D structure of the most similar F2D inhibitor compared to lorlatinib (Tc = 0.41).

Figure 10 represents the 2D structures of 9 macrocycles having a QED score ≥ 0.7. All 2D structures obtained are listed in the Supplementary Information. By visual inspection, we found that one compound seemed to be very close to lorlatinib, even if its Tanimoto coefficient (Tc) is only 0.41 (Figure 11).

#### 2.4.5. F2D Website

F2D was created to help researchers (medicinal chemists or biologists) find new protein kinase inhibitors. Thus, we provide an online interface to facilitate the use of F2D. This interface is freely accessible at http://frags2drugs.icoa.fr (accessed on 14 January 2026). F2D needs several input data: a protein kinase structure, a seed structure, and, optionally, the definition of atoms for growing. This leads to four initial steps required to launch F2D, detailed in a user guide. Firstly, the user has to select the desired target from its protein gene name (e.g., BRAF). A table of the PDB ID structures corresponding to this protein gene name is listed, and the user can choose the desired target structure. Finally, the user chooses a seed and selects the atoms for growing. The four selection steps are provided with a user-friendly interface.

**Figure 12:**
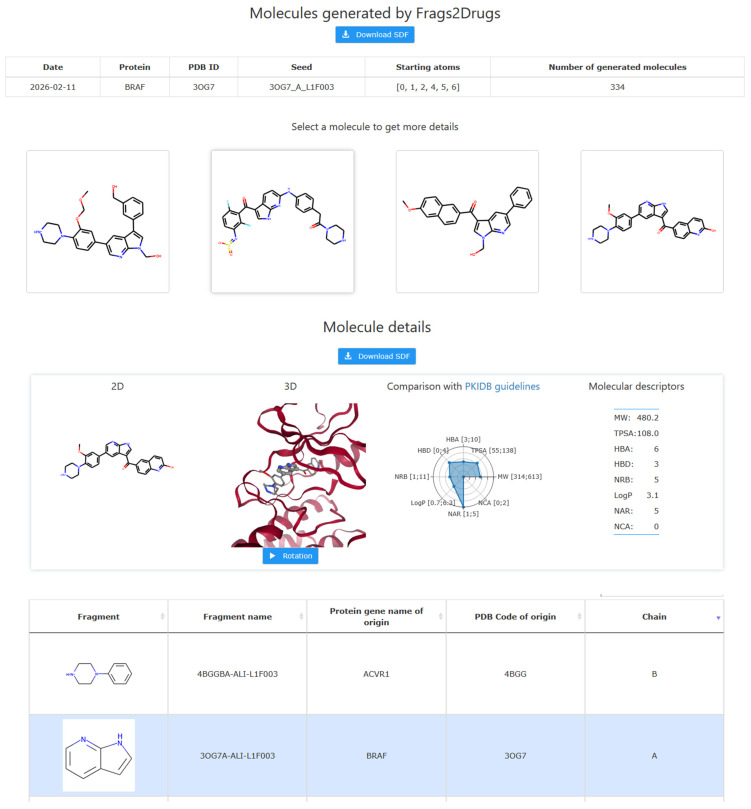
Example of results obtained from the Frags2Drugs website. This figure shows 4 of the 334 created molecules. On the website, all the molecules are available, displayed in an 8-by-8 matrix. The table showing the details for each fragment used in the selected molecule is also provided.

The search for new molecules starts. F2D will then provide a web page link to retrieve results. Once available, the results are displayed as shown in Figure 12. Furthermore, the parameters used and the number of generated molecules is detailed in a table. The 2D representation of built molecules is displayed in an 8-by-8 matrix. The user may select a molecule to get additional details. Results are downloadable as an SDF file to allow researchers to further process them. All results obtained on the F2D website are accessible for one month.

## 3. Discussion

We present here Frags2Drugs (F2D), a new in silico fragment-based ligand design tool built on a 3D fragment network and a growing-linking approach to find novel protein kinase inhibitors. Several PKI-like filters are applied, and a synthetic accessibility score is provided at the end of the process. We have demonstrated its success on several protein kinase examples for which we generated type I and type II inhibitors. In addition, we presented an example of finding novel macrocyclic inhibitors for the ALK protein kinase. Interestingly, we were able to identify compounds similar to known active molecules or drugs. The main advantage of F2D is the use of 3D experimental structures without the need for energy minimization after fragment growing and linking, because it uses empirical rules of the MMFF94 force field when creating covalent bonds. Furthermore, no systematic cavity exploration is needed; thus, the results can be rapidly obtained. F2D is freely accessible at http://frags2drugs.icoa.fr/ (accessed on 14 January 2026). Once the user has selected the target of interest, the seed, and the anchoring points, the tool provides a set of novel molecules bound inside the active site of the target.

F2D is able to create type I, type I1/2, and type II inhibitors of protein kinases, but can also generate more complex molecules such as macrocycles. Further study is ongoing to apply F2D to covalent kinase inhibitors.

F2D currently focuses on the design of protein kinase inhibitors. A great improvement would be to broaden the application of F2D to any protein family. Because F2D is based on the sequence and structure alignment of protein kinase-ligand complexes, this method cannot be applied to other protein families. In order to expand our application, several procedures may be envisaged, such as aligning cavities from different protein families [78] or moving fragments from a binding site to another using subpockets [79] or shape-based descriptors [80].

A key element of the creation of an F2D database is the sequence and structure alignment of all protein kinases so that the binding sites of crystallized structures are well superimposed. The seed position needs to be defined and positioned into the binding site to enable the creation of type I, type I1/2, and type II inhibitors. Therefore, allosteric inhibitors outside the active site cannot be created by F2D. Again, F2D could be improved by adding more fragments in allosteric sites, by using docking methods of fragments, or by repositioning fragments from similar subpockets.

The web interface of F2D is restricted to proteins with available crystallographic structures. The entire protein kinase space could be covered by using bioinformatics methods such as homology or AI modeling to predict the 3D structure of protein kinases.

The fragmentation of molecules is another key point of database creation. However, despite the use of published fragmentation algorithms to achieve this step, several known inhibitors could not be reconstructed because of these issues. We thus have to improve the implemented fragmentation methods to overcome this limitation. This could be achieved by rewriting our own method or using other established methods such as DAIM [81] or Ftrees-FS [82].

We have implemented a filtering procedure to reduce the number of molecules given by F2D, based on structural and physicochemical parameters of compounds. However, the predicted bioactivity of the created molecules is not currently considered. To guide the choice of the most interesting compounds, it could be wise to implement a deep learning model to predict binding affinities [83].

## 4. Materials and Methods

### 4.1. Protein Kinase Superimposition and Fragment Dataset

PDB ID of protein kinases was retrieved by using a request based on specific PFAM [84] identifiers: the PFAM id “PF07714” for protein tyrosine kinase and the PFAM id “PF00069” for protein kinase domain. These PFAM accession numbers gave us access to 3809 structures, regardless of the organism’s origin (July 2016). These structures were then split by chain using Biopython tools [85] (version 1.72). Structures having alternative locations for a few atoms were duplicated to treat cases independently. The alignment of the protein kinases on the reference protein kinase with the PDB ID 1ATP chain E was performed with Molecular Operating Environment (MOE, Chemical Computing Group, Montreal, QC, Canada, 2016_0802) [86].

Several algorithms already implemented in KNIME [87] were used to fragment the ligands: RECAP [88], BRICS [89], and Scaffold Tree [90]. The three methods were used to provide a larger number of different fragments, so further novel molecules will be created. Fragments were then standardized using VSPrep [91], a KNIME workflow. At this stage, a filter was applied to fragments. Fragment having a MW > 300 Da, a phosphate, a carbohydrate, an ester, an acyclic N-N, N-O, or O-O substructure was removed because of potential pharmacokinetic issues. The next step consisted of removing duplicate fragments. Here, we considered a fragment as a chemical group in a particular 3D orientation. So, duplicates were not only 2D identical compounds, such as SMILES, but also compounds having both the same SMILES and 3D identical coordinates. To manage this, we grouped the same fragments together, in clusters, based on their SMILES. Then, for each cluster, the full RMSD matrix was calculated between all members. For each pair of fragments having an RMSD value less than 0.25 Å, only the first one was kept. The database, at the end, contained several 3D orientations for the fragments.

Starting from 6204 initial ligands, the database finally contained 72,480 fragments. Each of these fragments was labeled with a unique identifier and information about its origin (PDB ID, chain, alternative location, Uniprot gene name, and species).

### 4.2. Creation of the 3D Fragment Network

We used Python (3.6.8), neo4j (3.5.11), and mainly the Python library networkx (2.2) to create the fragment network.

Calculation of the compatibility relationship was performed in two steps. Firstly, for each protein, its binding site cavity and the surrounding atoms were identified using FPocket [92], an open-source cavity detection tool based on Voronoï tessellation. Secondly, a fragment was defined as compatible with a cavity if the distances between its atoms and the atoms of the protein ranged between 1.4 Å and 3.5 Å. Thus, we avoided both steric hindrances with the fragment and the protein and built compounds far from the active site.

When two fragments were linked, the four calculated distortion parameter values ranged from 0 to 45, with 0 meaning there is no distortion compared to the reference value of the force field. For angles, increasing the value by one means authorizing a difference of one additional degree, while for distance, the value corresponded to a percentage of allowed deviation from the reference value of the force field, so the maximum distortion allowed for a distance was 45% of the reference value.

### 4.3. Molecular Generation and Filtering Process

Nodes from the graph database were associated with corresponding structures of fragments with the use of Pandas dataframe (1.0.5) and RDKit [93] (2020.03.3). RDKit was also used to link fragments together to generate molecules.

During the filtering process, substructure recognition relies on SMARTS patterns [94] and was applied by substructure match from RDKit. The PKI-like filter removed molecules containing undesired physicochemical parameters.

The molecular docking step was carried out with rDock [95] (2013.1) software. Before proceeding to docking, molecules were standardized and protonated. New conformations were generated using the RDKit method of conformation generation ETKDG [96] coupled to MMFF94 force field refinement [34]. New conformations allowed us to avoid the bias consisting of giving the docking software a conformation similar to that provided by F2D. Root Mean Square Deviation (RMSD) between molecules from F2D and docking poses was calculated with RDKit. We chose a threshold of 2.5 Å from the minimal RMSD values observed by applying the same procedure on all protein kinase-ligand complexes. Because rDock failed in generating conformations of macrocycles, the conformational search was performed with the MOE (Chemical Computing Group, Montreal, Quebec, Canada, 2019_0101) suite [97]. SA score calculation and PAINS substructure removal were achieved using RDKit modules. CNS MPO is calculated using ChemAxon’s Calculator plugins, Marvin 15.2.9, 2015, ChemAxon (https://www.chemaxon.com), and RDKit.

Similarity searches performed on whole databases used FPSim2 [98]. The similarity search was based on the Tanimoto coefficient [99] with a similarity threshold of 0.7. The versions of the four databases were ChEMBL 27 (accessed June 2020), ZINC 15 (accessed June 2020), Ambinter February 2020, and PKIDB 9 December 2020 http://www.icoa.fr/pkidb/ (accessed on 14 January 2021).

### 4.4. Validation of F2D Methodology

From 6204 initial ligands, we first selected the 4338 PKI-like ligands and removed staurosporine-like molecules (PDB ligand IDs STU, UCN, and LY4). Indeed, these compounds contain an indolocarbazole, which is not fragmented by the algorithms. Due to its MW > 300 Da, this group is too heavy to be kept in the database. We also removed the ligands having chemical issues in their registered structures in the PDB. This led to 3218 ligands being reconstructed to validate the F2D method.

For each of the 3218 ligands, we selected its corresponding nodes and edges from the graph database, and we applied F2D using each fragment alternatively as a seed. After each generation of molecules, we checked the presence of the original ligand among the molecules created. If the ligand was not found, we repeated the process using another seed.

### 4.5. F2D Website

F2D is freely accessible from http://frags2drugs.icoa.fr (accessed on 14 January 2026). This website is based on several Docker 20.10.5 containers communicating through docker-compose 1.28.6. The Python web framework Django LTS 2.2 and Celery 4.4.6 are used to run the website and F2D calculations in a task queue. The database relies on PostgreSQL 12.2 and the RDKit cartridge 2020.03.

### 4.6. Discovery of Kinase Inhibitors

The 3D position of the seeds used in the four examples (ABL, BRAF, MELK, and ALK) was obtained by removing all atoms, except those of the seed, from the co-crystallized molecules.

The four distortion parameters used while growing molecules in the examples presented in this article are shown in Table 2. The values vary depending on the number of generated molecules. If one wants to generate more molecules, the distortion parameters will be increased.

During the search for macrocycles, no restriction was applied on the distortion parameters to maximize the number of results.

## Figures and Tables

**Table 1:** PKI-like physicochemical properties used to filter molecules built by Frags2Drugs (F2D).

	Lower Limit	Upper Limit
MW (Da)	314	613
TPSA (Å^2^)	55	138
ClogP	0.7	6.3
HBA	3	10
HBD	0	4
NRB	1	11
NAR	1	5
NCA	0	2

MW: molecular weight; TPSA: topological polar surface area; HBA: hydrogen bond acceptors; HBD: hydrogen bond donors; NRB: number of rotatable bonds; NAR: number of aromatic rings; NCA: Number of chiral atoms.

**Table 2:** Distortion parameters used in F2D during fragment growing examples.

	Torsion Angle (deg)	Out of Plane Angle (deg)	Dihedral Angle (deg)	Distance (%)
BCR-ABL	15	15	15	10
BRAF V600E	10	10	10	10
MELK Type I	10	10	10	10
MELK Type II	14	14	14	12
ALK	10	10	10	10

## Data Availability

The original contributions presented in this study are included in the article and Supplementary Material. Further inquiries can be directed to the corresponding authors.

## Supplementary Materials

The following supporting information can be downloaded at: https://www.mdpi.com/article/10.3390/ph19020308/s1, Figure S1: Example of F2D applied to BRAF V600E (PDB ID 3OG7, chain A); Figure S2: Structures of the 15 molecules with the highest QED score generated by F2D on BRAF V600E from the pyrrolo[2,3-b]pyridine moiety seed; Figure S3: Example of F2D to the discovery of MELK type I inhibitors (PDB ID 4UMQ, chain A); Figure S4: Structures of the 9 predicted type I inhibitors, having the highest QED scores, generated by F2D on MELK from a benzamide seed; Figure S5. Example of F2D execution to discover type II inhibitors on MELK (PDB ID 4UMT, chain A); Figure S6. Structures of the 6 type II inhibitors, having the highest QED score, generated by F2D on MELK from a 3-(isoquinolin-7-yl)prop-2-yn-1-ol moiety seed. The sdf files contain the 50, 510, 41, 8 and 153 molecules found by F2D for BCR-ABL, BRAF V600E, MELK (type I), MELK (type II), and ALK respectively. The mol2 files of the proteins, the sdf files of co-crystallized ligands and the seeds are provided.

## Abbreviations

The following abbreviations are used in this manuscript:

ALK	Anaplastic Lymphoma Kinase
BBB	Blood–Brain Barrier
BRAF	B–Rapidly Accelerated Fibrosarcoma
BCR-ABL	Breakpoint Cluster Region–Abelson oncogene
ClogP	Calculated logP
CNS MPO	Central Nervous System Multiparameter Optimization
CML	Chronic Myeloid Leukemia
FBDD	Fragment-Based Drug Design
F2D	Frags2Drugs
Ge	Graph of exclusions
Gi	Graph of inclusions
rIFN-α	interferon-alfa
KLIFS	Kinase–Ligand Interaction Fingerprints and Structure database
Ro5	Lipinski’s Rule of Five
MELK	Maternal Embryonic Leucine zipper Kinase
MOE	Molecular Operating Environment
MW	Molecular Weight
NMR	Nuclear Magnetic Resonance
NAR	Number of Aromatic Rings
NCA	Number of Chiral Atoms
HBA	Number of Hydrogen Bond Acceptors
HBD	Number of Hydrogen Bond Donors
NRB	Number of Rotatable Bonds
OOP	Out of Plane
PAINS	Pan-Assay Interference Compounds
PDB	Protein DataBank
PKIDB	Protein Kinase Inhibitor Database
PKI	Protein Kinase Inhibitors
QED	Quantitative Estimate of Druglikeness
RCSB	Research Collaboratory for Structural Bioinformatics
RMSD	Root Mean Square Deviation
SAR	Structure Activity Relationships
SA	Synthetic Accessibility
3D	Three Dimensional
TPSA	Topological Polar Surface Area
FDA	U.S. Food and Drug Administration
