# Supplementary material for: Frags2Drugs: A Novel In Silico Fragment-Based Approach to the Discovery of Kinase Inhibitors
Source: Pharmaceuticals (Basel). 2026 Feb 12;19(2):308. doi: 10.3390/ph19020308 (PMC12944305; doi:10.3390/ph19020308)
Supplement: Supplementary file 1 [file pharmaceuticals-19-00308-s001.zip › pharmaceuticals-4126567-supplementary/SupplementaryData_bonnet/Supplementary Materials.pdf]

SUPPLEMENTARY INFORMATION

Frags2Drugs: A Novel in Silico Fragment-Based Approach to the Discovery of Kinase Inhibitors

Gautier Peyrat †, Colin Bournez †, Pascal Krezel, José-Manuel Gally, Stéphane Bourg, Samia Aci-Sèche * and Pascal Bonnet *

Université d’Orléans, CNRS, ICOA, UMR 7311, Orléans, France; gautier.peyrat@univ-orleans.fr (G.P.); colin.bournez@univ-orleans.fr (C.B.); pascal.krezel@univ-orleans.fr (P.K.); jose-manuel.gally@univ-orleans.fr (J.-M.G.); stephane.bourg@cnrs.fr (S.B.)

* Correspondence: samia.aci-seche@univ-orleans.fr (S.A.-S.); pascal.bonnet@univ-orleans.fr (P.B.);
Tel.: +33-2-38-41-99-02 (S.A.-S.); +33-2-38-41-72-54 (P.B.)

† These authors contributed equally.

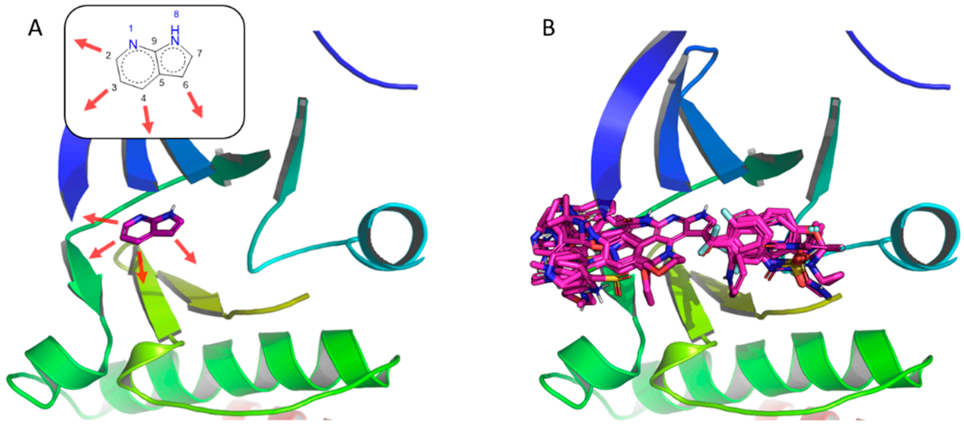

Figure S1. Example of F2D applied to BRAF V600E (PDB ID 3OG7, chain A). (A) Initial position of the seed from co-crystallized ligand, the red arrows indicate starting atoms and growing directions. (B) All of the 510 molecules obtained in the active site of BRAF V600E.

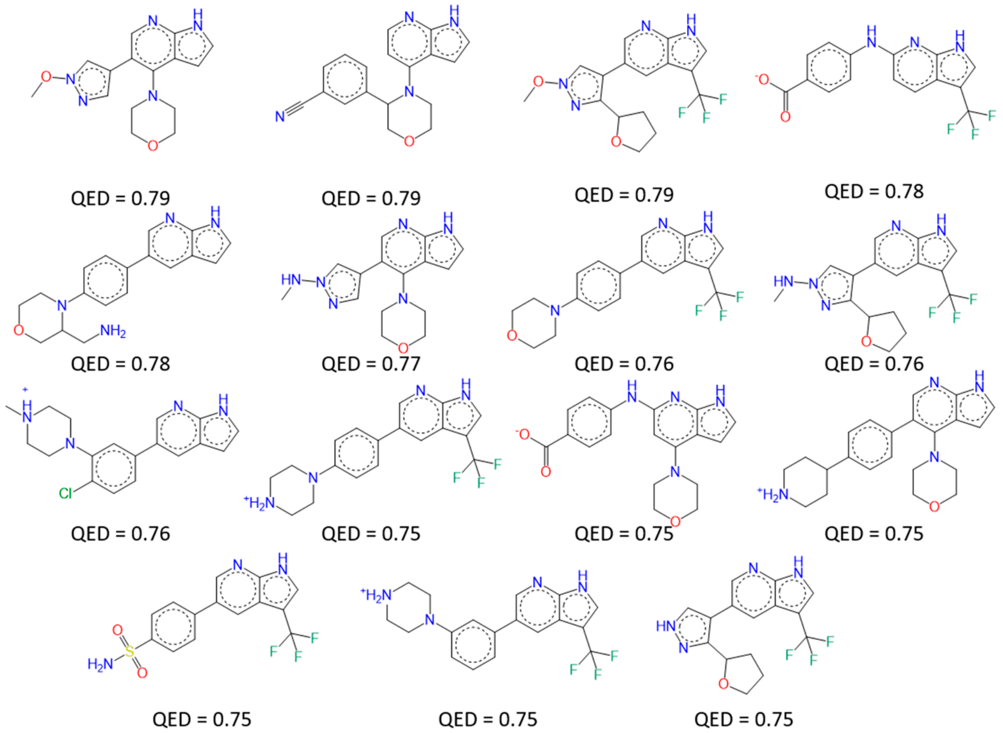

Figure S2. Structures of the 15 molecules with the highest QED score generated by F2D on BRAF V600E from the pyrrolo[2,3-b]pyridine moiety seed.

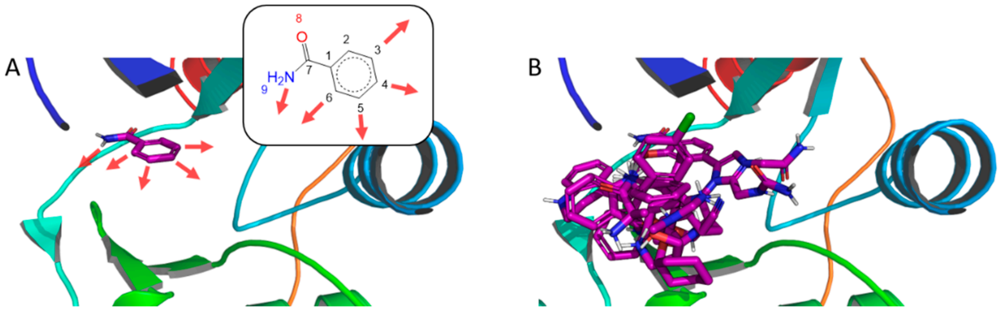

Figure S3. Example of F2D to the discovery of MELK type I inhibitors (PDB ID 4UMQ, chain A). (A) Initial position of the benzamide seed, the red arrows indicate starting atoms and growing directions. (B) All of the new molecules obtained superimposed in the active site of MELK.

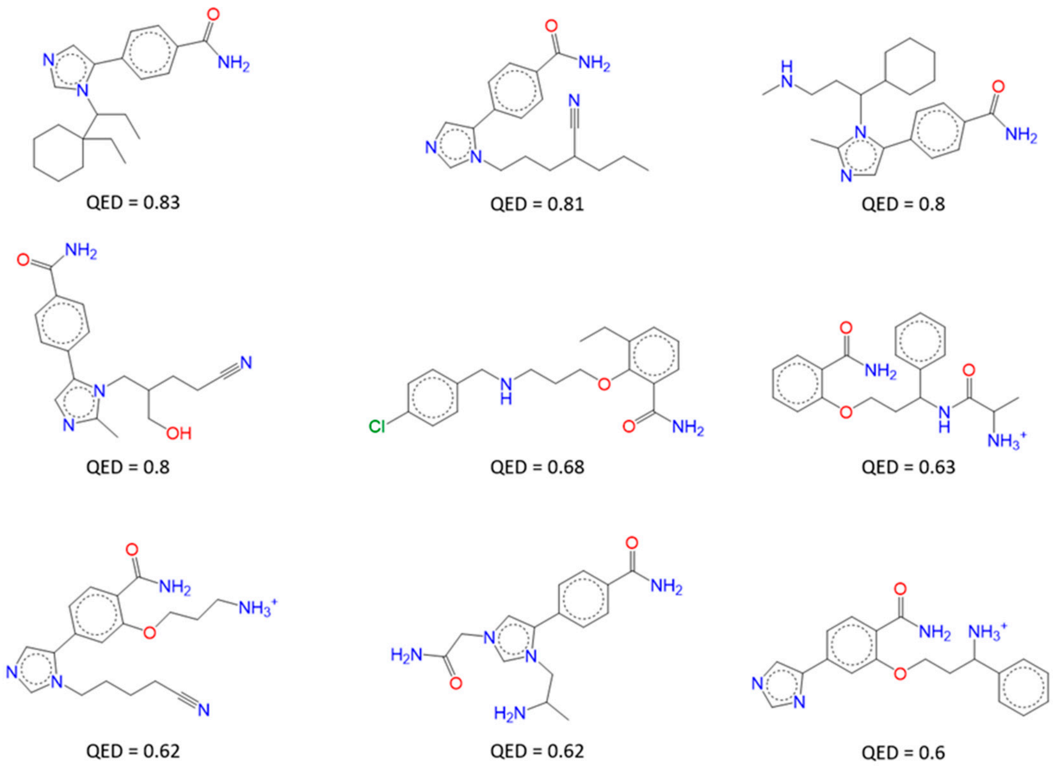

Figure S4. Structures of the 9 predicted type I inhibitors, having the highest QED scores, generated by F2D on MELK from a benzamide seed.

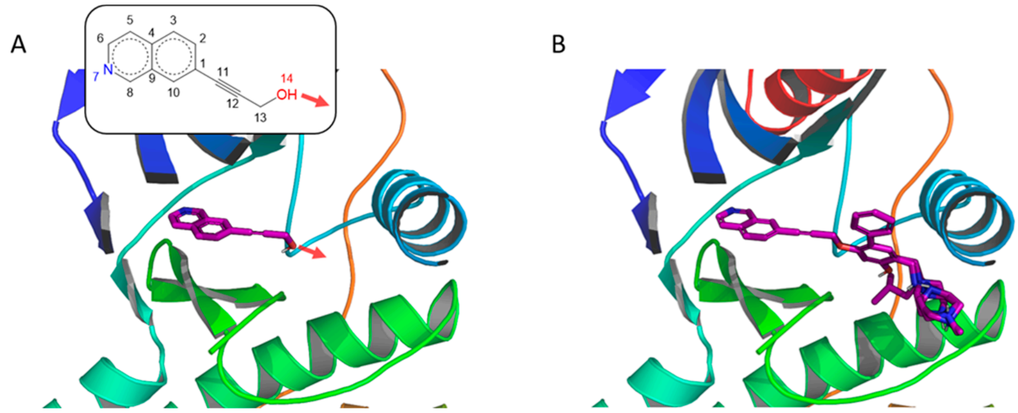

Figure S5. Example of F2D execution to discover type II inhibitors on MELK (PDB ID 4UMT, chain A). (A) Initial position of the 3-(isoquinolin-7-yl)prop-2-yn-1-ol moiety seed, the red arrow indicates the starting atom and the direction where to grow. (B) Eight new molecules were obtained, superimposed in the active site of MELK.

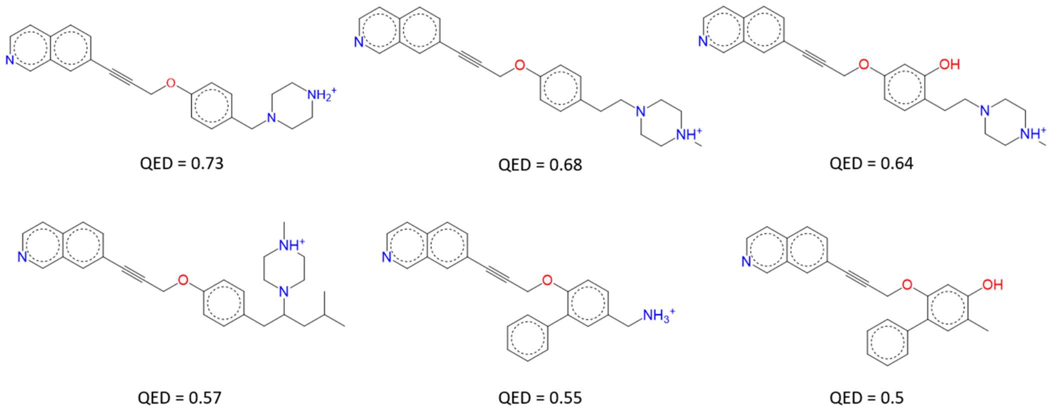

Figure S6. Structures of the 6 type II inhibitors, having the highest QED score, generated by F2D on MELK from a 3-(isoquinolin-7-yl)prop-2-yn-1-ol moiety seed.
